# Validity of diagnoses, procedures, and birth records in a Japanese administrative claims database for pediatric patients

**DOI:** 10.1111/ped.70178

**Published:** 2025-09-04

**Authors:** Wakana Maki, Hiroki Kitaoka, Shotaro Aso, Sachiko Ono, Motohiro Kato, Hideo Yasunaga

**Affiliations:** ^1^ Department of Clinical Epidemiology and Health Economics, School of Public Health The University of Tokyo Tokyo Japan; ^2^ Department of Pediatrics The University of Tokyo Tokyo Japan; ^3^ Department of Health Services Research, Graduate School of Medicine The University of Tokyo Tokyo Japan; ^4^ Department of Eat‐loss Medicine, Graduate School of Medicine The University of Tokyo Tokyo Japan

**Keywords:** administrative claims, data accuracy, electronic health records, healthcare, pediatrics, sensitivity and specificity

## Abstract

**Background:**

Administrative claims data are used in clinical studies; however, recorded diagnoses and procedures have not been fully validated for pediatric patients. We aimed to examine the validity of recorded information on pediatric patients in the Japanese Diagnosis Procedure Combination (DPC) database, a national inpatient database that includes administrative claims data.

**Methods:**

We validated the DPC data using medical charts as the reference standard. We included patients aged <16 years admitted to a single academic hospital in Tokyo between 2018 and 2022. Positive predictive values were assessed for six diagnoses (cardiac arrest, blood cancer, acute myocarditis, nontraumatic intracranial hemorrhage, hypoplastic left heart syndrome, and trisomy 21) and in‐hospital death. We evaluated intubation, mechanical ventilation, and high‐flow nasal cannulas in 100 randomly selected patients from the neonatal intensive care unit (NICU) and pediatric intensive care unit, and the gestational age and birth weight in NICU patients.

**Results:**

The positive predictive values of the diagnoses ranged from 70% (non‐traumatic intracranial hemorrhage) to 100% (trisomy 21 and in‐hospital death). The sensitivities of the procedures ranged from 36% (high‐flow nasal cannula in the NICU) to 89% (mechanical ventilation in the NICU), and the specificities were all >95%. The mean gestational ages were 34.2 weeks in the chart and 33.8 weeks in the claims, and the mean birth weights were 2101 g in the chart and 2104 g in the claims, demonstrating high concordance.

**Conclusion:**

Japanese claims data can be useful for pediatric studies focusing on selected diagnoses, procedures, and birth records with confirmed validity.

## INTRODUCTION

There is often a lack of solid evidence in pediatric patients. Ethical concerns often hinder the possibility of conducting interventional studies. Therefore, real‐world data collected from daily medical practice, including electronic health records and administrative data, are useful in pediatric clinical observational studies.^1^

The Diagnosis Procedure Combination (DPC) data in Japan consist of healthcare administrative claims data and an inpatient discharge summary, including data on patient characteristics (e.g., age and sex), diagnoses, procedures, and medications. This database is often used in clinical studies.^2^, ^3^ However, the data are not recorded for the purpose of clinical research but for reimbursement by public health insurance. Thus, studies that quantify the validity of the recorded information are essential.^4^, ^5^

Although many validation studies for administrative claims data have been conducted, studies in pediatric patients are scarce worldwide.^6^ Findings from validation studies on adult patients cannot be applied to pediatric patients because clinical characteristics vary with patient age.^6^, ^7^ Moreover, the administrative claims data depend on the health insurance system in each country. The findings of validation studies in foreign countries are not generalizable.^5^ Therefore, an original validation study of administrative claims data for pediatric patients in Japan is crucial.

However, the study populations of the most validation studies in Japan included only adults.^8^, ^9^, ^10^, ^11^, ^12^, ^13^, ^14^ Some studies did not exclude pediatric patients but did not describe in detail the number of included child patients.^15^, ^16^, ^17^, ^18^, ^19^, ^20^, ^21^, ^22^, ^23^, ^24^ A previous study included <10 child patients.^25^ To the best of our knowledge, only one study specifically on pediatric patients has elucidated the positive predictive value (PPV) of diagnostic codes for congenital malformation.^26^ Therefore, validation studies that focus exclusively on pediatric populations and on diseases and procedures that are considered clinically important in pediatric care are lacking.

In this study, we aimed to verify the validity of diagnosis and procedure codes, as well as gestational age at birth and birth weight in the DPC data of pediatric patients in Japan. We focused on diagnostic codes for high‐risk conditions in pediatric intensive care settings, frequently used respiratory support procedures, and key perinatal information. Given that diagnosis and procedure codes are frequently used in real‐world data research, including in algorithms that combine multiple codes to identify specific clinical conditions, establishing their validity is essential as a prerequisite for such applications.^4^ To achieve this aim, a record of a given diagnosis or procedure in medical charts can be considered sufficient to confirm the corresponding codes in claims data.^27^ This study provides foundational data that will support the appropriate use of Japanese administrative claims data in future pediatric research.

## METHODS

### Data source

This study was conducted using the electronic medical records and DPC data stored at the University of Tokyo Hospital. Diagnoses at admission and discharge were recorded using the International Classification of Diseases, Tenth Revision (ICD‐10) codes. All procedures performed during hospitalization were recorded using the original codes provided by the Ministry of Health, Labour and Welfare.^24^ Chart reviews were used as reference standards to evaluate the validity of the DPC data, including the sensitivity, specificity, PPV, and negative predictive value (NPV).

### Study participants

We included all patients aged <16 years admitted to the Department of Pediatrics at the University of Tokyo Hospital between April 2018 and March 2022. Of these, we randomly selected 100 patients hospitalized in the neonatal intensive care unit (NICU) and 100 in the pediatric intensive care unit (PICU). Hospitalizations in the NICU or PICU were identified using codes for intensive care unit management fees.

In addition to 200 randomly selected patients hospitalized in the NICU or PICU, patients who were diagnosed with the following six diseases or died during hospitalization were identified using the DPC data. Up to 100 patients with each diagnosis or in‐hospital mortality were identified. When the number of eligible patients exceeded 100, 100 patients were randomly selected.

This target sample size was based on the following considerations: First, according to a previous methodological guidline, to estimate a PPV with a 95% confidence interval of ±10%, a sample of approximately 100 cases is sufficient,^28^ and several prior validation studies have adopted the target of 100 cases per code based on this principle.^24^, ^29^, ^30^ Second, considering the feasibility of chart review within the available time and resources, we determined that a target sample size of 100 cases was practical.

### Diagnoses and in‐hospital death

The six diseases evaluated were cardiac arrest, blood cancer (malignant neoplasms of lymphoid, hematopoietic, and related tissues), acute myocarditis, nontraumatic intracranial hemorrhage, hypoplastic left heart syndrome, and trisomy 21. Five diseases, except for trisomy 21, were selected with reference to conditions identified as high‐risk in the Pediatric Index of Mortality 2 (PIM2), a mortality prediction model widely used in pediatric intensive care settings.^31^ In addition, we included trisomy 21 as one of the common genetic disorders.^32^ These conditions were identified based on ICD‐10 codes recorded at the time of admission and discharge, as detailed in Table S1. Diagnoses corresponding to multiple ICD‐10 definitions were grouped together and evaluated as a single diagnosis. We did not consider the severity or disease stage at each diagnosis.

### Procedures

Of the hospitalized patients in the NICU and PICU, three procedure codes were evaluated: (1) intubation (except planned intubation during surgeries or examinations); (2) mechanical ventilation with intubation, nasal continuous positive airway pressure (NCPAP), or nasal intermittent positive pressure ventilation (NIPPV) under defined conditions; and (3) high‐flow nasal cannula (HFNC). In Japan, the procedure codes recorded for medical reimbursement assign the same code (J045) to cases involving mechanical ventilation via endotracheal intubation, the use of NCPAP or intermittent mandatory ventilation (IMV) for neonates without endotracheal intubation, and the use of nasal mask ventilation for acute respiratory failure characterized by a partial pressure of oxygen (PaO_2_)/fraction of inspired oxygen (FiO_2_) ratio of ≤300 mmHg or a partial pressure of carbon dioxide (PaCO_2_) level of ≥45 mmHg. Consequently, it was impossible to differentiate between mechanical ventilation with intubation, NCPAP, and NIPPV based solely on these codes.

We evaluated whether patients underwent these three procedures on each day for a total of 6 days: the day of admission to the fifth day of hospitalization and the day before discharge. This approach assumes that respiratory support therapies are initiated early during hospitalization and is designed to simplify the chart review process. To observe the procedures that continued until discharge, the day before discharge was also included in the analysis. If hospitalization lasted <6 days, the first day of admission and the day before discharge were prioritized for recording. For example, if hospitalization lasted only one day, only the first day was recorded. For the 3‐day hospitalization, the first, second, and day before discharge were recorded.

The codes of all procedures performed during hospitalization were recorded along with the corresponding dates of implementation. However, the reimbursement policy in Japan has allowed only one ventilation support procedure to be recorded per day. Therefore, when patients receive both HFNC and other types of mechanical ventilation, such as intubation with mechanical ventilation, NCPAP, or NIPPV, a procedure with a higher reimbursement value, typically mechanical ventilation, has been more likely to be recorded.

### Gestational age at birth and birth weight

Among patients hospitalized in the NICU, gestational age at birth (recorded in weeks) and birth weight were also assessed.

### Chart reviews

Chart reviews were conducted at the hospital between January and November 2024. Two board‐certified pediatricians (W.M. and H.K.) independently conducted chart reviews of the cases and identified death, the presence of a diagnosis of each disease and each procedure, the gestational age, and birth weight. For each diagnosis group defined by multiple ICD‐10 codes, chart reviewers evaluated whether a diagnosis within the group had been established, rather than verifying the accuracy of individual ICD‐10 code assignments.

In this chart review, when a patient received multiple assisted ventilation therapies on the same day, we recorded whether the patient received both, regardless of the procedure coding policy in the claims data, which allowed only one type of respiratory therapy to be recorded per day. Disputes between the reviewers were resolved through discussion.

### Statistical analysis

Continuous variables are expressed as medians and interquartile ranges (IQRs), and categorical variables are expressed as numbers and percentages (%).

The inter‐reviewer agreements for the diagnoses, procedures, and in‐hospital death before the discussion were evaluated using kappa coefficients and categorized as follows: near‐perfect (0.81–1.00), substantial (0.61–0.80), moderate (0.41–0.60), fair (0.21–0.40), or poor (0.00–0.20).^8^, ^13^, ^24^ The sensitivity, specificity, PPV, and NPV were calculated for all three procedures. In all cases, claims data were considered to match with chart records when the procedure was documented in the claims data on the day of recording, as performed in the chart. These metrics were calculated for all assessed days combined (from the first to the fifth day of hospitalization and the day before discharge) as overall indicators for “all days,” and additionally, for each individual day separately.

The PPV was calculated for the diagnoses of the six diseases and in‐hospital deaths. The mean absolute difference and intraclass correlation coefficients were used to compare the gestational age (weeks) and birth weight between the claims data and chart records. Bland–Altman plots are presented for gestational age and birth weight.

In addition, we evaluated the validity of mechanical ventilation with intubation. The claims data were defined as having an intubation code recorded between the first and fifth days of hospitalization, followed by a mechanical ventilation code on or after that day. We focused on the implementation of mechanical ventilation with intubation during the first 5 days of hospitalization without considering the exact procedure dates.

This study was conducted in accordance with the principles of the Declaration of Helsinki. The study protocol was approved by the Ethics Review Board of the University of Tokyo (2023123NI), and written informed consent was not required. An announcement about the study and the possibility that participants could opt out of the study were made on the hospital's website.

## RESULTS

**FIGURE 1:**
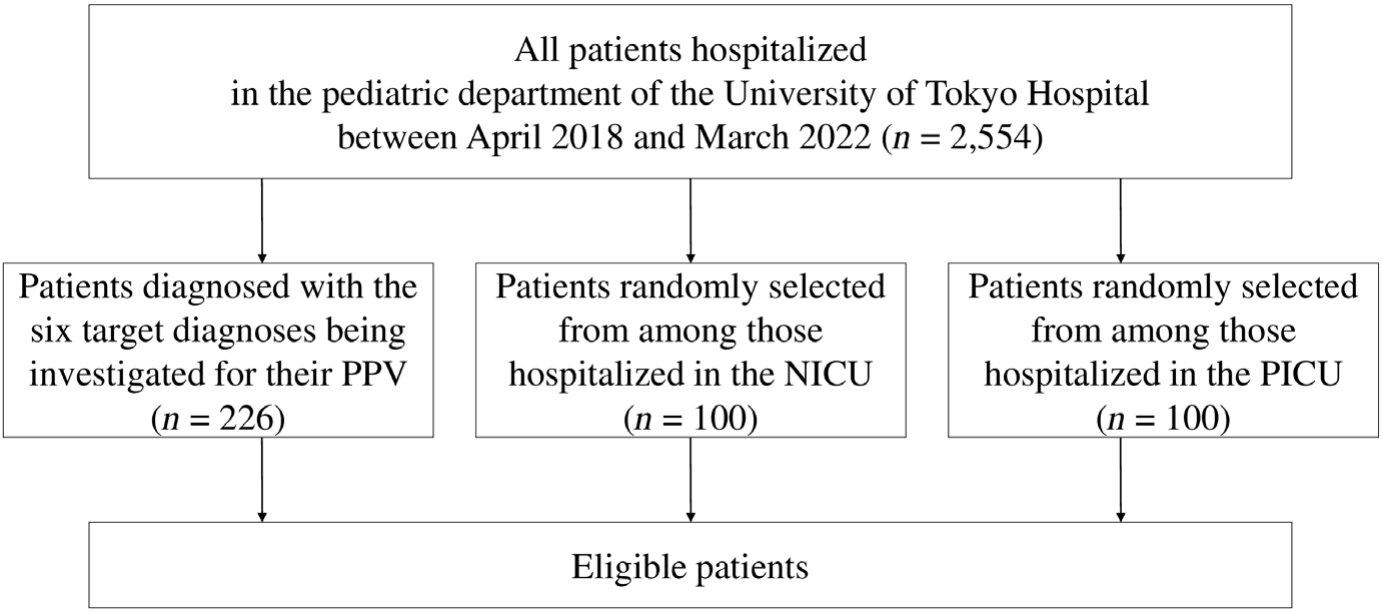
Patient flow. NICU, neonatal intensive care unit; PICU, pediatric intensive care unit; PPV, positive predictive value.

We identified 2554 patients admitted to the Department of Pediatrics between April 2018 and March 2022. We randomly extracted data from 100 patients hospitalized in the NICU and 100 patients hospitalized in the PICU (Figure 1). The numbers of patients with diagnoses of the above six diseases or in‐hospital death were 15 for cardiac arrest; 80 for malignant neoplasms of lymphoid, hematopoietic, and related tissues; 10 for acute myocarditis; 10 for nontraumatic intracranial hemorrhage; 22 for hypoplastic left heart syndrome; 53 for trisomy 21; and 36 for in‐hospital death.

Patient characteristics are shown in Table 1. The median age of the 216 patients included in the diagnosis name validation was 3 (IQR, 0–6) years. Of these, 53.5% (121/226) were male. In addition, the median age of the patients in the NICU was 0 (IQR, 0–0) days, and 52% (52/100) were male. The median age of the patients in the PICU was 1 (IQR, 0–3) year, and 45% (45/100) were male. The number of procedures performed daily for the 100 patients hospitalized in the NICU and PICU is shown in Tables 2 and 3.

**TABLE 1:** Patient characteristics.

Variables	Patients whose diagnoses were evaluated^a^ (*n* = 226)	Patients in the NICU (*n* = 100)	Patients in the PICU (*n* = 100)
Age in years, median (IQR)	3 (0–6)	‐	1 (0–3)
Age in days, median (IQR)	‐	0 (0–0)	‐
Male, *n* (%)	121 (53.5)	52 (52.0)	45 (45.0)

^a^ When a single patient has multiple diagnoses, each diagnosis is counted as one individual case. *N* = 226 indicates cumulative count.

**TABLE 2:** Frequencies of diagnoses and validity indices for claims‐based procedure identification in pediatric patients admitted to NICUs.

Procedures in the NICU	Eligible patients (*n*)	Frequency (charts), *n* (%)	Inter‐reviewer agreement (kappa)	Frequency (claims), *n* (%)	Sensitivity (95% CI)	Specificity (95% CI)	PPV (95% CI)	NPV (95% CI)
Intubation								
All days	584	36 (6.2)	0.96	24 (4.1)	0.64 (0.46–0.79)	1.00 (0.99–1.00)	0.96 (0.79–1.00)	0.98 (0.96–0.99)
Day 1	100	32 (32.0)	1.00	21 (21.0)	0.66 (0.47–0.81)	1.00 (0.95–1.00)	1.00 (0.84–1.00)	0.86 (0.77–0.93)
Day 2	99	2 (2.0)	1.00	2 (2.0)	0.50 (0.01–0.99)	0.99 (0.94–1.00)	0.50 (0.01–0.99)	0.99 (0.94–1.00)
Day 3	97	1 (1.0)	0.00	0 (0)	‐	‐	‐	‐
Day 4	95	0 (0)	1.00	0 (0)	‐	‐	‐	‐
Day 5	94	1 (1.1)	0.00	1 (1.1)	1.00 (0.25–1.00)	1.00 (0.96–1.00)	1.00 (0.03–1.00)	1.00 (0.96–1.00)
Day before discharge	99	0 (0)	1.00	0 (0)				
Mechanical ventilation with intubation, or CPAP or NIPPV								
All days	584	273 (46.7)	0.96	247 (0.42)	0.89 (0.84–0.92)	0.98 (0.96–1.00)	0.98 (0.95–0.99)	0.91 (0.87–0.94)
Day 1	100	55 (55.0)	0.98	45 (0.45)	0.80 (0.67–0.90)	0.98 (0.88–1.00)	0.98 (0.88–1.00)	0.80 (0.67–0.90)
Day 2	99	57 (57.6)	0.98	55 (55.6)	0.97 (0.88–1.00)	1.00 (0.92–1.00)	1.00 (0.94–1.00)	0.96 (0.85–0.99)
Day 3	97	56 (57.7)	0.92	54 (55.7)	0.93 (0.83–0.98)	0.95 (0.84–0.99)	0.96 (0.87–0.99)	0.91 (0.78–0.97)
Day 4	95	52 (54.7)	1.00	45 (47.4)	0.87 (0.74–0.94)	1.00 (0.92–1.00)	1.00 (0.92–1.00)	0.86 (0.73–0.94)
Day 5	94	48 (51.1)	0.91	41 (43.6)	0.85 (0.72–0.94)	1.00 (0.92–1.00)	1.00 (0.92–1.00)	0.87 (0.75–0.95)
Day before discharge	99	5 (5.1)	0.65	7 (7.1)	1.00 (0.48–1.00)	0.98 (0.93–1.00)	0.71 (0.29–0.96)	1.00 (0.96–1.00)
HFNC								
All days	584	36 (6.2)	0.99	18 (3.1)	0.50 (0.33–0.67)	1.00 (0.99–1.00)	1.00 (0.82–1.00)	0.97 (0.95–0.98)
Day 1	100	6 (6.0)	0.90	4 (4.0)	0.67 (0.22–0.96)	1.00 (0.96–1.00)	1.00 (0.40 1.00)	0.98 (0.93–1.00)
Day 2	99	5 (5.1)	0.90	5 (5.1)	1.00 (0.48–1.00)	1.00 (0.96–1.00)	1.00 (0.48–1.00)	1.00 (0.96–1.00)
Day 3	97	8 (8.2)	0.75	3 (3.1)	0.38 (0.09–0.76)	1.00 (0.96–1.00)	1.00 (0.29–1.00)	0.95 (0.88–0.98)
Day 4	95	9 (9.5)	0.94	2 (0.2)	0.22 (0.03–0.60)	1.00 (0.96–1.00)	1.00 (0.16–1.00)	0.93 (0.85–0.97)
Day 5	94	8 (8.5)	1.00	4 (4.3)	0.50 (0.16–0.84)	1.00 (0.96–1.00)	1.00 (0.40–1.00)	0.96 (0.89–0.99)
Day before discharge	99	0 (0)	‐	0 (0)	‐	‐	‐	‐

**TABLE 3:** Validities of gestational age at birth (in week) and birth weight (gram) for the claims‐based identification.

	Eligible patients	Charts	Claims	Difference	ICC (chart claim)
	*n*	Mean (SD)	Mean (SD)	Mean (95% CI)	ICC (95% CI)
Gestational age at birth (in week)	99^a^	34.2 (4.7)	33.8 (5.8)	0.4 (−1.1–0.4)	0.85 (0.78–0.90)
Birth weight (gram)	99^a^	2101 (878)	2104 (880)	−2.9 (−8.4–2.5)	1.00 (1.00–1.00)

Abbreviations: CI, confidence interval; ICC, intraclass correlation coefficient; SD, standard deviation.

^a^ The gestational age and birth weight of one patient were missing from the claims.

### Validity of diagnoses

Table 4 shows the frequencies, inter‐reviewer agreements, and PPVs of the six diseases and in‐hospital deaths. The inter‐reviewer agreement on the diagnoses of hypoplastic left heart syndrome, trisomy 21, and in‐hospital death among hospitalized patients during chart review was nearly perfect, even prior to discussions between the reviewers. In contrast, the incidences of cardiac arrest, acute myocarditis, and nontraumatic intracranial hemorrhage were moderate. Malignant neoplasms of the lymphoid, hematopoietic, and related tissues were fairly common. The PPV ranged from 70.0% (nontraumatic intracranial hemorrhage) to 100% (trisomy 21 and in‐hospital death).

**TABLE 4:** Frequencies of diagnoses s and validity indices s for claims‐based diagnostic identification in pediatric patients.

Diagnoses	Eligible patients (*n*)	Frequency (charts) (*n*)	Inter‐reviewer agreement (kappa)	Frequency (claims)	PPV (95% CI)
Cardiac arrest	15	13	0.63	15	0.87 (0.62–0.96)
Malignant neoplasms of lymphoid, hematopoietic, and related tissues	80	77	0.36	80	0.96 (0.90–0.99)
Acute myocarditis	10	8	0.62	10	0.80 (0.49–0.94)
Nontraumatic intracranial hemorrhage	10	7	0.55	10	0.70 (0.40–0.89)
Hypoplastic left heart syndrome	22	19	0.83	22	0.86 (0.67–0.95)
Trisomy 21	53	53	1.00	53	1.00 (0.93–1.00)
In‐hospital death	36	36	1.00	36	1.00 (0.90–1.00)

### Validity of procedures

Tables 2 and 5 show the frequencies, inter‐reviewer agreements, and validity indicators for the three procedures. For the NICU patients, the inter‐reviewer agreements for intubation, mechanical ventilation with intubation, NCPAP, NIPPV, and HFNC across all recorded days were nearly perfect. For PICU patients, the inter‐reviewer agreement for mechanical ventilation with intubation, NCPAP, and NIPPV across all recorded days was nearly perfect, whereas that for intubation and HFNC was substantial. The sensitivity of mechanical ventilation was high in both NICU and PICU patients (89% and 79%, respectively). This was followed by an intubation sensitivity of 64% in the NICU and 58% in the PICU. The sensitivity of HFNC was the lowest among the three procedures, at 50% in the NICU and 36% in the PICU. For both NICU and PICU patients, the specificities of the three procedures were all >95%.

**TABLE 5:** Frequencies of diagnoses and validity indices for claims‐based procedure identification in pediatric patients admitted to PICUs.

Procedures in the PICU	Eligible patients (*n*)	Frequency (charts) *n* (%)	Inter‐reviewer agreement (kappa)	Frequency (claims) *n* (%)	Sensitivity (95% CI)	Specificity (95% CI)	PPV (95% CI)	NPV (95% CI)
Intubation								
All days	507	24 (4.7)	0.68	26 (5.1)	0.58 (0.37–0.78)	0.98 (0.96–0.99)	0.54 (0.33–0.73)	0.98 (0.96–0.99)
Day 1	100	19 (19.0)	0.84	18 (18.0)	0.63 (0.38–0.84)	0.93 (0.85–0.97)	0.67 (0.41–0.87)	0.92 (0.83–0.97)
Day 2	89	4 (4.5)	0.40	4 (4.5)	0.50 (0.07–0.93)	0.98 (0.92–1.00)	0.50 (0.07–0.93)	0.98 (0.92–1.00)
Day 3	79	0	0	2 (2.5)	‐	‐	‐	‐
Day 4	75	1 (1.3)	0	2 (2.6)	0.00 (0.00–0.98)	0.97 (0.91–1.00)	0.00 (0.00–0.84)	0.99 (0.93–1.00)
Day 5	69	0	0	0	‐	‐	‐	‐
Day before discharge	95	0	1.00	0	‐	‐	‐	‐
Mechanical ventilation with intubation, or CPAP or NIPPV								
All days	507	202 (39.8)	0.91	169 (33.3)	0.77 (0.70–0.82)	0.95 (0.92–0.98)	0.92 (0.87–0.95)	0.86 (0.82–0.90)
Day 1	100	36 (36.0)	0.98	33 (33.0)	0.81 (0.64–0.92)	0.94 (0.85–0.98)	0.88 (0.72–0.97)	0.90 (0.80–0.96)
Day 2	89	49 (55.1)	0.89	38 (42.7)	0.74 (0.59–0.85)	0.95 (0.83–0.99)	0.95 (0.82–0.99)	0.75 (0.60–0.86)
Day 3	79	43 (54.4)	0.95	37 (46.8)	0.84 (0.69–0.93)	0.97 (0.86–1.00)	0.97 (0.86–1.00)	0.83 (0.69–0.93)
Day 4	75	35 (46.7)	0.89	31 (41.3)	0.80 (0.63–0.92)	0.93 (0.80–0.98)	0.90 (0.74–0.98)	0.84 (0.70–0.93)
Day 5	69	29 (42.0)	0.82	21 (30.4)	0.66 (0.46–0.82)	0.95 (0.83–0.99)	0.91 (0.70–0.99)	0.79 (0.65–0.90)
Day before discharge	95	10 (10.5)	0.79	9 (9.5)	0.70 (0.35–0.93)	0.98 (0.92–1.00)	0.78 (0.40–0.97)	0.97 (0.90–0.99)
HFNC								
All days	507	47 (9.3)	0.73	22 (4.3)	0.36 (0.23–0.52)	0.99 (0.98–1.00)	0.77 (0.55–0.92)	0.94 (0.91–0.96)
Day 1	100	8 (8.0)	0.75	3 (3.0)	0.38 (0.09–0.76)	1.00 (0.96–1.00)	1.00 (0.30–1.00)	0.95 (0.88–0.98)
Day 2	89	8 (9.0)	0.64	3 (3.4)	0.38 (0.09–0.76)	1.00 (0.96–1.00)	1.00 (0.30–1.00)	0.94 (0.88–0.98)
Day 3	79	7 (8.9)	0.63	3 (3.8)	0.29 (0.04–0.71)	0.99 (0.93–1.00)	0.67 (0.09–0.99)	0.93 (0.85–0.98)
Day 4	75	10 (13.3)	0.70	6 (0.08)	0.40 (0.12–0.74)	0.97 (0.89–1.00)	0.67 (0.22–0.96)	0.91 (0.82–0.97)
Day 5	69	8 (11.6)	0.84	3 (4.3)	0.25 (0.03–0.65)	0.98 (0.91–1.00)	0.67 (0.09–0.99)	0.91 (0.81–0.97)
Day before discharge	95	6 (6.3)	0.82	4 (4.2)	0.50 (0.12–0.88)	0.99 (0.94–1.00)	0.75 (0.19–0.99)	0.97 (0.91–0.99)

In an additional analysis that examined the validity of mechanical ventilation with intubation by combining the intubation and mechanical ventilation codes, the sensitivity and specificity of mechanical ventilation with intubation during the first 5 days of hospitalization were 63% and 100% in the NICU and 38% and 100% in the PICU, respectively (Table S2).

### Validity of gestational age at birth (in weeks) and birth weight (grams) of neonatal intensive care unit patients

The gestational age at birth (weeks) and birth weight (g) of the NICU patients in the charts and claims are shown in Table 3. The mean (standard deviation [SD]) of gestational age at birth in the chart review was 34.2 (4.7) weeks, whereas the mean (SD) of that in the claims was 33.8 (5.8). The mean (SD) birth weight in the chart review was 2101 (878) g, whereas the mean (SD) birth weight in the claims was 2104 (880) g. The Bland–Altman plots for gestational week at birth and birth weight are presented in Figures S1 and S2, respectively. Only one pair of gestational week values and two pairs of birth weight values differed between the claims data and chart review. These discrepancies were not associated with a specific gestational age or birth weight.

## DISCUSSION

This study evaluated the validity of Japanese claims data regarding diagnoses and procedures during hospitalization in the NICU or PICU, gestational age at birth, and birth weight of patients hospitalized in the NICU recorded in the claims data, with chart reviews serving as the gold standard.

### Validity of diagnoses

The PPV of the diagnosis was >80%, except for nontraumatic intracranial hemorrhage (70%). Notably, the rates of trisomy 21 and in‐hospital deaths were 100%. The reason for the low PPV for nontraumatic intracranial hemorrhage was unclear. This may be attributed to previous diagnosis records that were retained in the claims, even when the condition resolved and the patient no longer had it. In contrast, diseases such as trisomy 21 may have higher PPVs because trisomy 21 can be definitively diagnosed and remains unchanged throughout life.

The discrepancies between the reviewers regarding the diagnosis of malignant neoplasms of lymphoid, hematopoietic, and related tissues stemmed from differing judgments on whether to classify patients in remission who were hospitalized for other conditions as having a diagnosis. However, following the reviewers' discussion, which considered patients in remission to have the disease, the PPVs reached a sufficiently high level of 90%. Our findings indicate that although the PPVs of diagnoses are high, accurately identifying patients in specific states, such as remission for long‐term conditions, such as blood cancer, requires more than diagnostic labels alone. Additional factors, such as treatment history, should also be considered.

Notably, our study demonstrated a PPV of 100% for in‐hospital deaths in children, defined based on the discharge status. This result exceeded the PPV reported in a previous study focusing on adults aged 65–74 years, in which the PPV for death at discharge was 95.7%^33^ The possible explanation for this is that child deaths are rare and critical outcomes, even in academic hospitals.

### Validity of procedures

The specificity of the procedures was consistently high among the patients hospitalized in the NICUs and PICUs. However, the sensitivities of intubation and HFNC in both the NICUs and PICUs were < 60%. A possible explanation for this is that the procedure code for intubation is designed specifically for cases of resuscitation, excluding its use in diagnostic or surgical procedures. However, in practice, intubation is occasionally performed for both resuscitation and other procedures. This discrepancy may result in some intubations being performed on the same day, as certain procedures were excluded from the coding as resuscitation‐related intubations. When the HFNC procedure is performed on the same day as other forms of mechanical ventilation, it may be underreported because of the coding policy, which allows only one respiratory support therapy to be coded per day. This can potentially reduce the sensitivity of the HFNC code.

Our additional analysis highlights that using both procedure codes for intubation and mechanical ventilation to identify patients who underwent mechanical ventilation with intubation more specifically is useful if low sensitivity is acceptable.

### Validity of gestational age at birth and birth weight

Both gestational age at birth (in weeks) and birth weight recorded in the claims data demonstrated high reliability compared with those recorded in the medical charts. Information on gestational age at birth and birth weight is essential for neonatal research. Evidence from perinatal research using the DPC database can be high because of the high validity of gestational age at birth and birth weight.

### Strengths and limitations

#### Strengths

To the best of our knowledge, this is the first study to examine the validity of the codes of diagnoses, procedures, as well as gestational age at birth, and birth weight in pediatric patients using claims data from Japan.

This study has the potential to benefit researchers conducting studies on pediatric patients using claims data in Japan, such as the DPC data. We demonstrated high PPVs for six diagnoses and in‐hospital mortality, high specificity for the three procedures in the claims data, and strong concordance for neonatal gestational age and birth weight.

Furthermore, we randomly selected patients hospitalized in the NICU and PICU, enabling a more robust validation study.

#### Limitations

First, it was conducted at a single academic hospital. In particular, the prevalence of each disease in academic hospitals may differ from that in the general population. Therefore, our findings may not be generalizable to other facilities. Second, a limited number of diagnoses was selected for validation. Common pediatric conditions, such as respiratory or gastrointestinal infections and external injuries, were not included in this study. Future research should address these disease categories, ideally in different healthcare settings. Third, we only evaluated the PPVs of diagnoses instead of sensitivities and specificities. Sensitivity and specificity estimations require not only confirmed cases but also a sufficiently large number of non‐cases to serve as a comparison group. Given the rarity of the diseases included in our dataset, assessing these metrics with adequate precision would have required a substantially larger number of patient records, which was not feasible. Therefore, we prioritized PPV as the most practical and appropriate measure for evaluating the accuracy of the given diagnostic codes.^27^ Fourth, multiple ICD‐10 codes representing the same disease category were grouped and evaluated as a single diagnosis, which may limit the granularity of the validation. Fifth, we evaluated the J045 procedure code, which includes cases of mechanical ventilation via intubation, CPAP, or IMV for neonates without intubation, and nasal mask ventilation for acute respiratory failure. However, chart reviews could not confirm whether the required conditions for J045 coding (PaO_2_/FiO_2_ ≤ 300 mmHg or PaCO_2_ ≥ 45 mmHg) were met.

## CONCLUSION

This study is the first to validate the diagnoses, procedures, gestational age at birth, and birth weight of pediatric patients in the Japanese claims data through chart reviews. Our findings suggest that Japanese claims data may be valuable for real‐world studies involving pediatric patients because of the high PPVs for the six diagnoses and in‐hospital mortality, high specificity for the three procedures, and strong concordance for neonatal gestational age and birth weight. However, further studies are needed to assess the validity of other diagnoses and procedures.

## AUTHOR CONTRIBUTIONS

WM conceived and designed the study. The study was supervised by SA, SO, MK, and HY. Data analyses were performed by WM and HK. The funding was provided by WM and HY. The first draft of the manuscript was written by WM, and all authors commented on previous versions of the manuscript. All the authors have read and approved the final version of the manuscript.

## FUNDING INFORMATION

This work was supported by grants from the Ministry of Health, Labour and Welfare, Japan (grant numbers: 23AA2003 and 22AA2006) and Japan Society for the Promotion of Science (JSPS) Grant‐in‐Aid for JSPS Fellows (grant number: JP24KJ0602).

## CONFLICT OF INTEREST STATEMENT

No one declared.

## ETHICS APPROVAL

This study was conducted in accordance with the principles of the Declaration of Helsinki. The study protocol was approved by the Ethics Review Board of the University of Tokyo (2023123NI), and written informed consent was not required. An announcement about the study and the possibility that participants could opt out of the study was made on the hospital's website.

## Supporting information


**Figure S1.** Discrepancy in reported gestational week at birth between claims data and chart review.

**Figure S2.** Discrepancy in reported birth weight at birth between claims data and chart review.

**Table S1.** The definitions of the six diagnoses and in‐hospital deaths for which positive predictive values were obtained in this study.
**Table S2.** Validity of mechanical ventilation with intubation by combining intubation and mechanical ventilation codes.

## Data Availability

The authors do not have permission to share data owing to reasons of sensitivity.
