# Supplementary figures and images for: Validity of diagnoses, procedures, and birth records in a Japanese administrative claims database for pediatric patients
Source: Pediatr Int. 2025 Sep 4;67(1):e70178. doi: 10.1111/ped.70178 (PMC12410039; doi:10.1111/ped.70178)

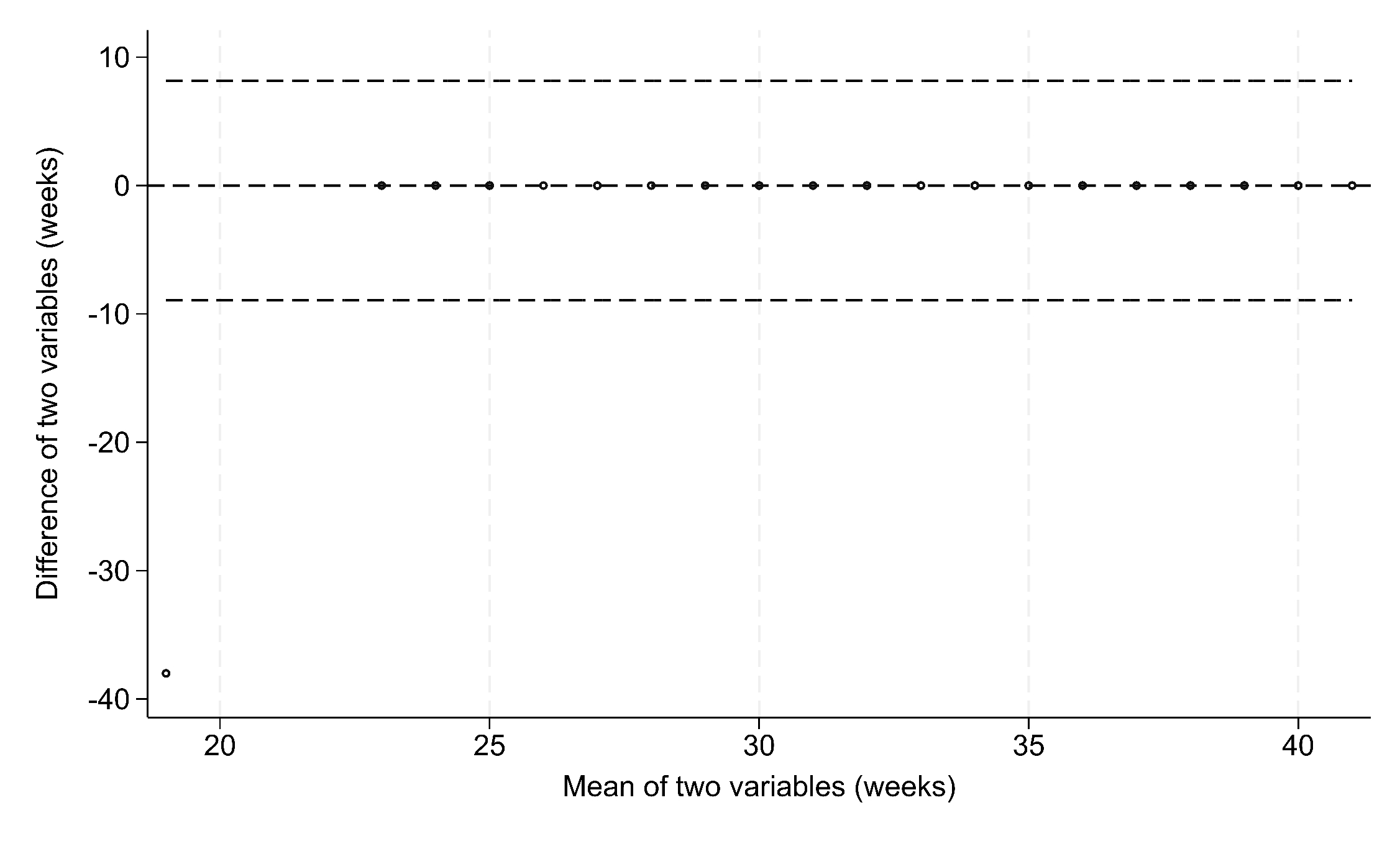

Supplement: Supplementary file 1 — Figure S1. Discrepancy in reported gestational week at birth between claims data and chart review. [file PED-67-e70178-s002.jpg]

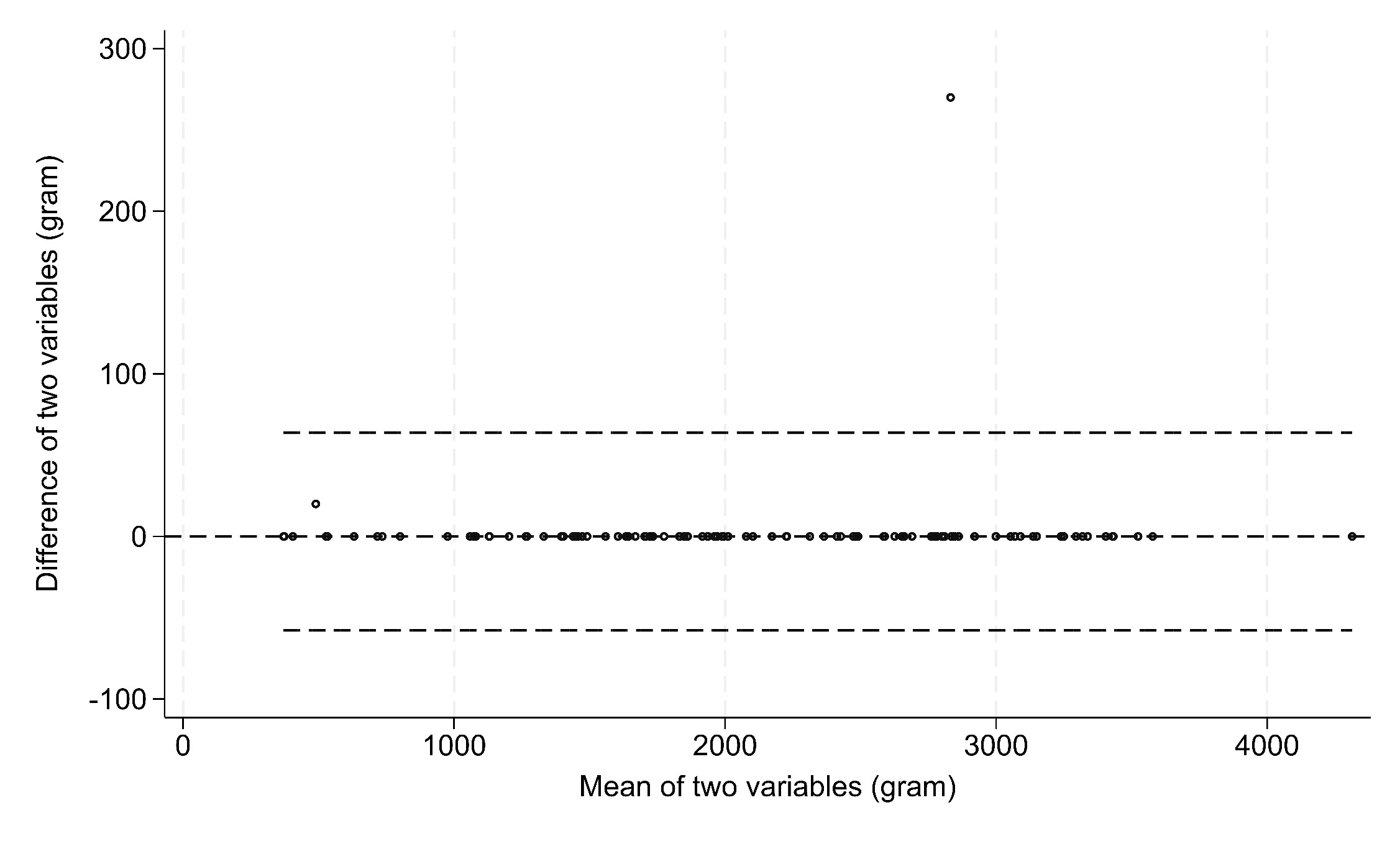

Supplement: Supplementary file 2 — Figure S2. Discrepancy in reported birth weight at birth between claims data and chart review. [file PED-67-e70178-s003.jpg]
