# Supplementary material for: Validity of diagnoses, procedures, and birth records in a Japanese administrative claims database for pediatric patients
Source: Pediatr Int. 2025 Sep 4;67(1):e70178. doi: 10.1111/ped.70178 (PMC12410039; doi:10.1111/ped.70178)
Supplement: Supplementary file 3 — Table S1. The definitions of the six diagnoses and in‐hospital deaths for which positive predictive values were obtained in this study. Table S2. Validity of mechanical ventilation with intubation by combining intubation and mechanical ventilation codes. [file PED-67-e70178-s001.docx]

Supplementary Table 1. The definitions of the six diagnoses and in-hospital deaths for which positive predictive values were obtained in this study

|  | Definitions (except for death): The inclusion of any of the following ICD-10 codes in the admission diagnosis. |
| --- | --- |
| **Diagnoses** |  |
| Cardiac arrest | I46.x (I46.0, I46.1, and I46.9) |
| Malignant neoplasms of lymphoid, hematopoietic, and related tissues | C81.x, C82.x, C83.x, C84.x, C85.x, C86.x, C88.x, C90.x, C91.x, C92.x, C93.x, C94.x, C95.x, C96.x |
| Acute myocarditis | I40.x |
| Nontraumatic intracranial hemorrhage | I60.x, I61.x, I62.x |
| Hypoplastic left heart syndrome | Q23.4 |
| Trisomy 21 | Q90.0, Q90.1, Q90.2, Q90.9 |
| In-hospital death | The outcome at discharge is recorded as death. |

^*^Diagnoses corresponding to multiple ICD-10 definitions were grouped together and evaluated as a single diagnosis.

Supplementary Table 2. Validity of mechanical ventilation with intubation by combining intubation and mechanical ventilation codes

|  | Eligible patients | Frequency  (charts) | Frequency  (claims) | Sensitivity (95% CI) | Specificity (95% CI) | PPV (95% CI) | NPV (95% CI) |
| --- | --- | --- | --- | --- | --- | --- | --- |
|  | n | n (%) | n (%) |  |  |  |  |
| **Procedures in the NICU** |  |  |  |  |  |  |  |
| Intubation + mechanical ventilation | 100 | 38 (38.0) | 24 (24.0) | 0.63 (0.46–0.78) | 1.00 (0.94–1.00) | 1.00 (0.86–1.00) | 0.82  (0.71–0.90) |
| **Procedures in the PICU** |  |  |  |  |  |  |  |
| Intubation + mechanical ventilation | 100 | 58 (58.0) | 23 (23.0) | 0.38 (0.26–0.52) | 0.98 (0.87–1.00) | 0.96 (0.78–1.00) | 0.53  (0.42–0.65) |

^*^We focused on the implementation of mechanical ventilation with intubation during the first 5 days of hospitalization without considering the exact procedure dates.
